# Acute Impact of Cancer Treatment on Head and Neck Cancer Patients: FIT4TREATMENT

**DOI:** 10.3390/cancers14112698

**Published:** 2022-05-30

**Authors:** Inês Leão, Catarina Garcia, Pedro Antunes, Ana Campolargo, Isabel Dias, Edite Coimbra, Pedro Oliveira, Horácio Zenha, Horácio Costa, Andreia Capela, Sofia Viamonte, Alberto J. Alves, Ana Joaquim

**Affiliations:** 1 Medical Oncology Department, Centro Hospitalar de Vila Nova de Gaia/Espinho Vila Nova de Gaia, 4434-502 Vila Nova de Gaia, Portugal; andreia.capela@gmail.com (A.C.); anaisabeljoaquim@gmail.com (A.J.); 2 ONCOMOVE^®^—Associação de Investigação de Cuidados de Suporte em Oncologia (AICSO), 4410-406 Vila Nova de Gaia, Portugal; catarina.garcia@ismai.pt (C.G.); pantunes_14@hotmail.com (P.A.); anacampolargo@gmail.com (A.C.); sofiaviamonte@gmail.com (S.V.); ajalves@ismai.pt (A.J.A.); 3 Research left in Sports Sciences Health Sciences and Human Development, University of Maia, 4475-690 Maia, Portugal; 4 Research left in Sports Sciences Health Sciences and Human Development, University of Beira Interior Covilhã, 6201-001 Covilhã, Portugal; 5 Physical Medicine and Rehabilitation Department, Centro Hospitalar de Vila Nova de Gaia/Espinho Vila Nova de Gaia, 4434-502 Vila Nova de Gaia, Portugal; 6 Nutrition Department, Centro Hospitalar de Vila Nova de Gaia/Espinho Vila Nova de Gaia, 4434-502 Vila Nova de Gaia, Portugal; idias@chvng.min-saude.pt; 7 Otorhinolaryngology Department, Centro Hospitalar de Vila Nova de Gaia/Espinho Vila Nova de Gaia, 4434-502 Vila Nova de Gaia, Portugal; editecoimbra@gmail.com (E.C.); pedrojorgeoliveira@gmail.com (P.O.); 8 Plastic Surgery Department, Centro Hospitalar de Vila Nova de Gaia/Espinho Vila Nova de Gaia, 4434-502 Vila Nova de Gaia, Portugal; hzcosta@sapo.pt (H.Z.); hcosta@chvng.min-saude.pt (H.C.)

**Keywords:** head and neck cancer, exercise training, quality of life, cognitive function, nutritional status, physical function

## Abstract

**Simple Summary:**

Head and neck cancer treatment causes toxicities that compromise health-related quality of life (HRQoL) and treatment efficacy. Exercise training (ET) benefits are reported for many cancer types. The aim of our prospective observational study was to analyse acute treatment’s impact and ET preferences. In the pretreatment phase (n = 18), most patients presented low physical function, were moderately malnourished or at risk of malnutrition, and were willing to participate in an ET program. Patients submitted to radical chemoradiotherapy (n = 7) experienced a significant decline in HRQoL and social functioning, an increase in dysphagia severity, a reduction in handgrip strength, and nutritional status deterioration. An ET program may optimize patients’ physical fitness, achieving more efficacy with less toxicity.

**Abstract:**

Head and neck cancer (HNC) treatment’s toxicities impact several health domains. Exercise training (ET) may be beneficial. This prospective observational study (NCT04996147) aimed to analyse the acute impact of HNC curative multimodal treatment on health-related quality of life (HRQoL), nutritional status, physical and cognitive functions, and ET preferences. Eighteen patients with stage III/IV HNC were evaluated at baseline (T0), and 10 patients were evaluated at the end of treatment (T1), 7 of them after radical chemoradiotherapy (rCRT). At T0, the majority referred a good HRQoL on the EORTC QLQ-C30 questionnaire (median score: 70.8), were moderately malnourished or at risk of malnutrition (78%), recognized the benefits of an ET program, and were willing to participate (78%). After rCRT, there was worsening in HRQoL (75 vs. 50 score, *p* = 0.014), dysphagia severity (Eating Assessment Tool: 7 vs. 31, *p* = 0.027; Functional Oral Intake Scale: 6 vs. 4, *p* = 0.041), handgrip strength (dominant: 40.9 vs. 35.8 kgf, *p* = 0.027; nondominant: 37.2 vs. 33.9 kgf, *p* = 0.043), and nutritional status (Patient-Generated Subjective Global Assessment: 7 vs. 18, *p* = 0.028). HNC patients subjected to radical treatment represent a vulnerable population that might benefit from multimodal supportive care strategies including an ET program.

## 1. Introduction

Head and neck squamous cell carcinoma (HNSCC) accounts for approximately 6% of all cancer cases and about 2% of deaths [1]. The majority of head and neck cancers (HNCs) originate in the oral cavity (44–55%), larynx (25–31%), or pharynx (16–25%) [1,2], and up to 90% have squamous cell histology [2]. At diagnosis, 33% of patients present localized disease (stages I–II), 50% locoregional involvement (stages III and IV with nodal metastasis), and about 10% distant metastasis [2].

Despite having locoregionally advanced disease, patients with stage III to IVB tumours can be treated with curative intent [2]. This treatment usually involves a multimodal approach with combinations of surgery, radiotherapy, and systemic chemotherapy [3,4], designed to balance the competing goals of tumour eradication and organ preservation [5]. Indeed, a curative outcome may be achieved in 70% to 95% of cases [5,6]. Nevertheless, up to two-thirds of patients have disease recurrence within the first 2 years of follow-up, and the 5-year survival rate ranges between 25% and 65%, depending on the precise primary site and stage [1].

Moreover, the aggressive nature of HNSCCs and their treatment modalities is associated with an important physical and psychological burden that persists into survivorship, which affects quality of life (i.e., malnutrition, difficulties in swallowing or speaking) [7,8], and enclosure-specific rehabilitation needs. This may be explained not only by the complex anatomy and vital role of the structures involving the tumour but also by the treatment’s acute and late toxicities that go beyond the adverse effects commonly reported by other cancer populations. For instance, more than 50% of patients treated with concomitant cisplatin (once every 3 weeks) and radiotherapy report grade 3+ toxicity, especially mucositis, often leading to temporary or definitive treatment interruption [9]. 

Despite the paramount importance of this topic, with a potential impact on the tumour response to treatment, recurrence, and survival, studies assessing the impact of cancer treatment on HNC patients’ well-being are lacking [8,10,11]. 

The benefits of exercise training (ET) have increasingly been recognized in cancer patients. In 2015, a systematic review and meta-analysis [12] reported a mean improvement of 5.55 points (*p* < 0.001) in quality of life (QoL) among cancer patients who enrolled in ET programs compared with normal care. However, no HNC patients were included in this study. Indeed, exercise clinical trials are still an emergent field in HNC. Nevertheless, some studies demonstrated that ET during treatment was feasible and might improve function and QoL [13,14,15]. Recently, Lin and colleagues observed improvements in body composition, muscle strength, and balance after 8 weeks of an ET protocol during CRT [15]. Moreover, significant differences were also observed in global health status, physical functioning, role functioning, emotional functioning, fatigue, appetite loss, feeling ill, and weight gain, favouring the intervention group [15]. This is noteworthy since general symptoms, such as fatigue and weight loss, have a significant impact on the HRQoL of HNC patients [16]. Therefore, this may suggest that the deterioration of these parameters during CRT can eventually be reversed with exercise. In addition, benefits of lean body mass have also been suggested [17,18], which is important, since low skeletal muscle mass is associated with treatment-related toxicities and poor overall survival [19,20]. The potential benefits of a physical exercise program as part of cancer treatment are just starting to be investigated, and they may change clinical practice [7,12,13,15,17,18,21].

To study this supportive care strategy in HNC patients, we designed a two-phase project (FIT4TREATMENT) comprising: (1) a prospective observation study to better understand the real impact of multimodal curative intent treatment on patients’ QoL, cognitive and physical function, and ET preferences and (2) a randomized controlled trial to test an ET program. Here, we present the results of phase 1.

## 2. Materials and Methods

### 2.1. Study Population

Eligible patients were more than 18 years old, diagnosed with stage III to IVB HNSCC, and proposed for one of the following strategies of multimodal treatment with curative intent: induction chemotherapy (CT) followed by surgery (which could be followed by radiotherapy), surgery followed by adjuvant chemoradiotherapy (CRT), and induction CT before CRT or radical CRT alone. The exclusion criteria included: synchronous tumours or other comorbidities with associated uncontrolled symptoms, an inability to provide informed consent, and an expected inability to fulfil the proposed schedule and follow-up.

**Figure 1:**
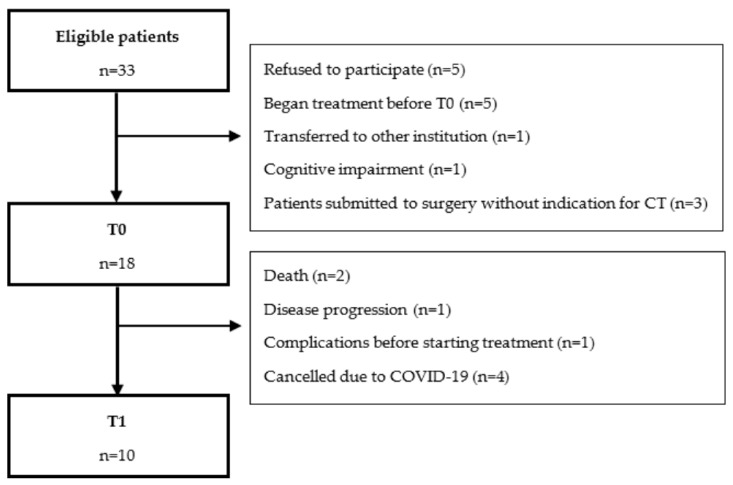
Consort diagram.

Recruitment started in June 2019, and due to the COVID-19 pandemic, the study was closed in March 2020, with 21 patients recruited (80.8% of the 26 patients presented at the multidisciplinary tumour board that fulfilled eligibility criteria). Three patients submitted to surgery were excluded because they did not fulfil the criteria for adjuvant concomitant chemoradiotherapy. Therefore, 18 patients were assessed at baseline (T0). No patients withdrew from the study. Ten patients completed the T1 evaluation (Figure 1).

All patients were male. The ages of the patients in our cohort ranged from a minimum of 44 years to a maximum of 75 years. Five patients (27.8%) were 65 years old or older. Most members of the study population were married, 50.0% had primary education or less, and 11.1% had higher education. Radical CRT was the most commonly prescribed treatment (50.0%). The sociodemographic and disease-related characteristics are presented in Table 1.

### 2.2. Design

This was a prospective observational study performed at Centro Hospitalar Vila Nova de Gaia/Espinho (CHVNG/E) in Portugal. Potential cases were identified by the multidisciplinary head and neck tumour board, and an informed consent form was signed upon eligibility. Included participants were scheduled for a baseline assessment before the beginning of treatment (T0) and at the end of treatment (T1).

Treatment strategies could include: induction chemotherapy (CT) with docetaxel 75 mg/m^2^, cisplatin 75 mg/m^2^, fluorouracil 750 mg/m^2^/day for 5 days (TPF) every 21 days for 3 cycles followed by surgery (which could be followed by radiotherapy), surgery followed by adjuvant radiotherapy (60–66 Gy) with concurrent cisplatin (100 mg/m^2^ every 21 days) for 6 to 6.5 weeks, induction CT with TPF every 21 days for 3 cycles before radiotherapy (70 Gy) with concurrent weekly cetuximab (initial dose of 400 mg/m^2^, followed by 250 mg/m^2^) for 6 weeks, or radical radiotherapy (70 Gy) with concurrent cisplatin (100 mg/m^2^ every 21 days) for 6 weeks. The time between T0 and T1 varied from 6 to 24 weeks depending on the treatment modality.

This study was conducted in compliance with the Declaration of Helsinki Ethical Principles (2013) and received approval by the Ethics Committee of CHVNG/E (reference number: 102/2019). The study is registered in clinical trials (NCT04996147).

### 2.3. Endpoints

#### 2.3.1. Health-Related Quality of Life

The primary endpoint was the global HRQoL score, assessed by the self-administered European Organisation for Research and Treatment of Cancer (EORTC) Quality of Life C-30 (QLQ-C30) questionnaire [22]. We also analysed other multi-item functional and symptom scales. Additionally, we used EORTC QLQ-H&N43 [23] to evaluate head-and-neck-cancer-specific items. Scores ranged from 0 to 100; in functional scales, high scores represent a high level of functioning, while in symptoms, they represent a high level of symptomatology.

#### 2.3.2. Nutritional Status and Body Composition

Nutritional status was assessed by a nutritionist using the Patient-Generated Subjective Global Assessment (PG-SGA) [24]. This validated and specific nutritional assessment tool allows a practical identification of malnutrition in cancer patients and triages necessary interventions [25,26]. Patients not only were categorized into one of three groups, well nourished (A), moderately malnourished (B), and severely malnourished (C), but also received a score from 0 to 35, with a higher score reflecting a greater risk of malnutrition (scores over 3 indicate a need for nutritional intervention). In addition, body weight, fat mass, and skeletal muscle mass were evaluated using bioelectrical impedance (Tanita BC-545). The body mass index (BMI) was also calculated (BMI = weight (kilograms)/height^2^ (meters)), and height was evaluated using Seca 220.

#### 2.3.3. Physical Function

Upper and lower body muscle strength was evaluated by maximal isometric handgrip and quadriceps strength, respectively, using two different dynamometers (Saehan Corporation, Masan, Korea, model SH5001, and The Advanced Force Gauge, Mecmesin). Subjects performed three trials in each arm and leg, and the mean value was recorded. Lower limb functionality was evaluated by the 30 s sit-to-stand test (STS). Additionally, functional capacity was assessed with the 6 min walk test (6MWT), which was standardized in accordance with the American Thoracic Society guidelines, taking into consideration the course length (20 m) [27].

#### 2.3.4. Dysphagia

The severity of dysphagia was assessed by the Eating Assessment Tool (EAT-10) [28] and Functional Oral Intake Scale (FOIS) [28]. The EAT-10 is a patient-reported outcome of self-perceived symptoms of dysphagia that was validated to assess initial oropharyngeal dysphagia and to identify changes in response to therapy. The total score ranges between 0 and 40, with a score ≥ 3 indicating dysphagia. The FOIS is a 7-item scale that reflects the functional oral intake of patients with dysphagia. Levels 1–3 relate to varying degrees of nonoral feeding, and levels 4–7 relate to degrees of oral feeding without nonoral supplementation.

#### 2.3.5. Cognitive Function

Cognitive function was assessed with the Montreal Cognitive Assessment (MoCA) [29]. MoCA is a cognitive screening test designed to assist the detection of mild cognitive impairment. Scores range between 0 and 30. A score of 26 or higher is considered normal.

#### 2.3.6. Exercise Training Preferences

A specific multiple-choice questionnaire was developed to understand patients’ preferences on ET before the beginning of treatment. It asked whether participants perceived possible exercise benefits, and enquired their interest in participating in an exercise program for HNC patients. Responders declaring an interest also answered questions regarding exercise preferences for frequency, intensity, duration, and type of exercise at three different time points, taking into consideration the timing of treatment (before, during, or after treatment). This questionnaire was first validated on a sample of 12 HNC patients to assess possible difficulties of interpretation, and the questions were adapted accordingly.

### 2.4. Statistical Analysis

The planned enrolment was 20 patients. Continuous variables were presented as median and interquartile range (IQR), and categorical variables as frequencies and percentage. Descriptive statistics of demographics and baseline (T0) characteristics of all the patients recruited were reported. A comparative analysis of endpoints measured before (T0) and after (T1) treatment was performed to assess the acute impact of CRT using the paired-sample Wilcoxon nonparametric test. Statistical data analysis was performed using the Statistical Package for the Social Sciences, version 26.

## 3. Results

### 3.1. Baseline Characterization

#### 3.1.1. Health-Related Quality of Life

Baseline EORTC QLQ-C30 and EORTC QLQ-HN43 results are presented in Table 2. At baseline, most patients reported a good global QoL (median score: 70.8 (IQR: 50.0–83.3)) and social function (median score: 100 (IQR: 66.7–100)). The most relevant HNC symptoms reported were dry mouth or sticky saliva (median score: 25 (IQR: 0–33.3)) and swallowing difficulty (median score: 20.8 (IQR: 0–50.0)). 

#### 3.1.2. Physical Function

The results of isometric handgrip strength were 38.0 kgf for the dominant hand and 37.1 kgf for the nondominant hand. Isometric quadriceps muscle strength was similar in dominant and nondominant limbs (31.7 kgf (IQR: 20.7–36.5) and 30.5 kgf (IQR: 20.9–35.6), respectively). The median 6MWT distance at baseline was 434 m (IQR: 399–533.8). Patients performed a median of 13.5 repetitions (IQR 12.0–15.5) in the 30 s sit-to-stand test.

#### 3.1.3. Nutritional Status and Body Composition

Six patients (33.3%) were severely malnourished, 8 (44.4%) were moderately malnourished or at risk of malnutrition, and 4 patients (22.2%) were identified as well nourished. Patients scored a median of 12.0 points in PG-SGA, which indicates a critical need for improved symptom management and/or nutrition intervention options. There was a positive correlation between PG-SGA total score and pain, evaluated in the EORTC QLQ-C30 questionnaire (ρ = 0.631, *p* = 0.005). 

The median weight at baseline was 60.7 kg (IQR: 51.4–69.5), with fat mass accounting for 19.3% (IQR: 11.3–24.1) of body mass and fat-free mass accounting for 50.7% (IQR: 43.8–53.2). The self-reported median weight in the previous month was 63.5 kg (IQR: 50.9–73.0). Considering the 11 patients that recalled their weight from 6 months prior, there was a median weight loss of 10.7% (IQR: 4.6–18.2). Of these patients, 6 (54.5%) reported a weight loss greater than 10%. The median BMI was 21.9 kg/m^2^ (IQR: 18.0–25.1), with 6 patients (33.3%) having a BMI below 18.5 kg/m^2^ (underweight) and 4 patients (22.2%) having a BMI above 25.0 kg/m^2^ (overweight). Six patients (33.3%) reported a decrease in their weight during the previous two weeks. The median BMI did not differ according to clinical stage (IVA vs. IVB: 21.1 kg/m^2^ (IQR: 17.9–25.4) vs. 21.4 kg/m^2^ (IQR: 19.2–27.4), *p* = 0.919); however, patients with stage IVA disease scored significantly less in PG-SGA compared with stage IVB (10.0 (IQR: 5.5–14.3) vs. 19.0 (IQR: 13.0–25.5), *p* = 0.019).

#### 3.1.4. Cognitive Function

The median MoCA score was 23 points (IQR: 20.8–26.3), and about 66.7% of the patients had a MoCA score below 26.

#### 3.1.5. Dysphagia

The median baseline EAT-10 score was 7.5 points (IQR: 1.8–22.3), with 13 patients (72.2%) scoring 3 or higher, an indication of abnormal swallowing function. According to FOIS, 3 patients (16.7%) had a total oral diet with no restrictions. Eleven patients (61.1%) had a total oral diet with multiple consistencies but requiring special preparation or compensations; 3 (16.7%) had a total oral diet not requiring special preparation but with specific food limitations; 3 (5.6%) had nothing by mouth. There was a strong inverse association between FOIS and EAT-10 (ρ = −0.748, *p* < 0.001). There was a strong association between PG-SGA total score and EAT-10 (ρ = 0.635, *p* = 0.005).

#### 3.1.6. Exercise Preferences

**Figure 2:**
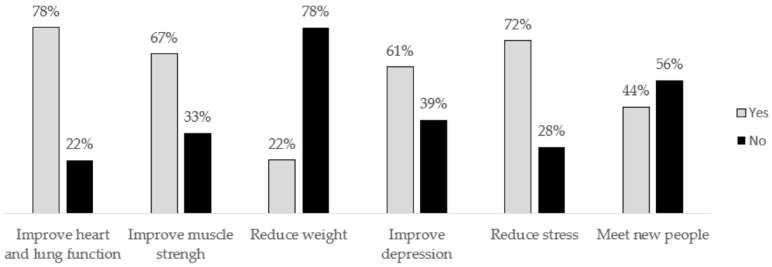
Possible benefits of ET program (n = 18).

Most patients recognized the possible benefits of participating in an ET program (Figure 2). The majority (77.8%) of these patients were willing to participate in an ET program before (35.7%), during (64.3%), and/or after (92.9%) treatment. 

**Figure 3:**
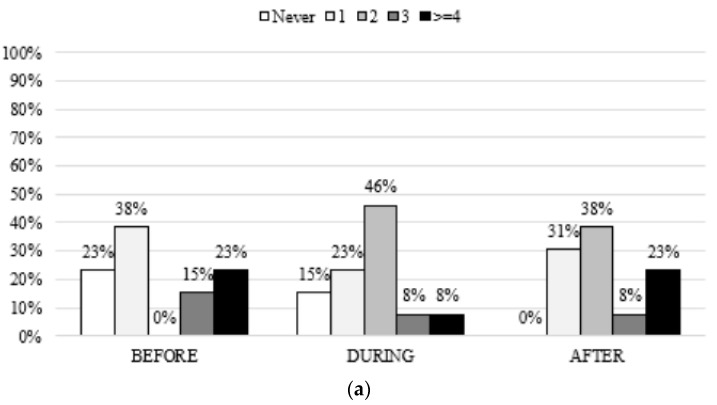

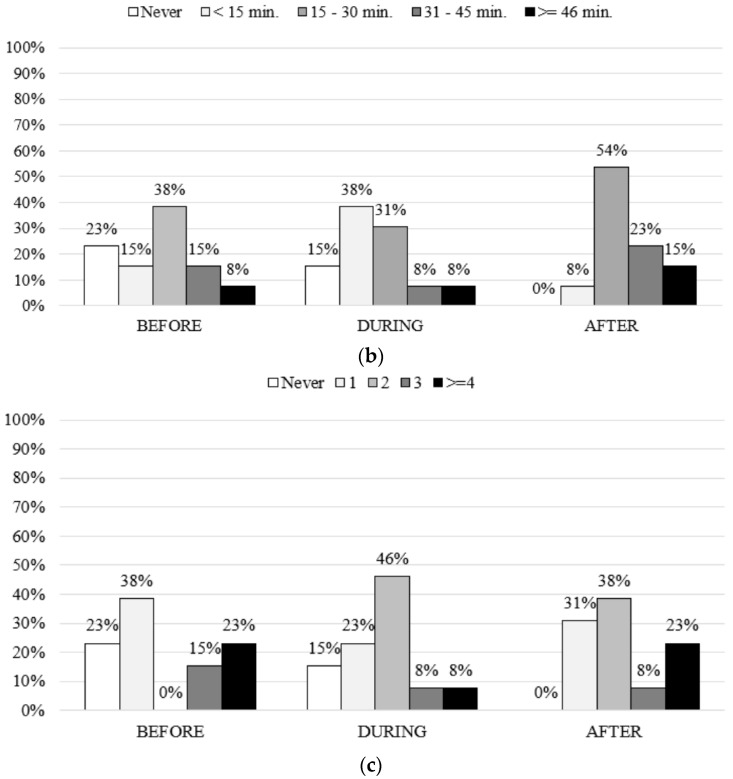
Exercise training preferences at baseline regarding (**a**) frequency (times/week), (**b**) duration, and (**c**) intensity.

The most common exercise preferences before and during treatment were at a frequency of one to two times/week, 15–30 min/bout at a light intensity. After treatment, patients reported an absolute increase in training frequency, duration, and intensity (most people were willing to participate in an ET program more than two times/week, at moderate intensity and 38.5% for more than 30 min (Figure 3)).

### 3.2. Acute Effects of Radical Chemoradiotherapy

Seven patients submitted to radical CRT completed the baseline (T0) and post-treatment (T1) assessment. Changes over time in HRQoL, physical function, nutritional status, dysphagia symptoms, and cognitive function are presented in Table 3.

After treatment, there was a significant reduction in global HRQoL (75.0 vs. 50.0 median score, *p* = 0.014) and social functioning (100 vs. 66.7 median score, *p* = 0.046), but no impact on emotional functioning. Despite the lack of impact on physical functioning, evaluated by the EORTC QLQ-C30 questionnaire, there was a significant reduction in handgrip strength on dominant (39.7 vs. 35.0 kgf, *p* = 0.018) and nondominant limbs (37.2 vs. 33.9 kgf, *p* = 0.043). Patients maintained quadriceps strength, distance covered on the 6MWT, and repetitions on the 30 s STS.

One patient was dependent on a feeding tube since the beginning of treatment. Of the six remaining patients, 83% experienced dysphagia and two needed percutaneous endoscopic gastrostomy tubes during treatment. Most patients recognized swallowing liquids as a severe problem, as well as associated pain and reduced eating pleasure (T1 FOIS median score: 4). Five patients (71%) had an EAT-10 score ≥ 3 before treatment, while at the end of CRT, all patients scored higher (7 vs. 31, *p* = 0.027). Scores of EAT-10 and FOIS were both strong and negatively correlated before (ρ = −0.982, *p* < 0.001) and after radical CRT (ρ = −0.833, *p* = 0.020).

At the end of treatment, 83.3% of the patients were severely malnourished, and all needed nutritional intervention (PG-SGA score ≥ 9). The median weight loss was 11.5% (IQR: 3.7–15.7%).

## 4. Discussion

Managing HNC patients comprises particular challenges. Because of the aggressiveness of cancer treatment, these patients experience high symptom burden and a decrease in QoL. Understanding the acute effects of the treatment and the impact of the disease itself is essential to improving the supportive care of these patients and, ultimately, increasing treatment tolerability and effectiveness.

Due to the improvement in cancer care with longer survival outcomes, QoL has become one of the most important concerns in oncology. Moreover, some studies suggest that QoL may be an independent prognostic factor in HNC patients [30,31,32]. In the literature, there is heterogeneity in the global QoL score reported by HNC patients [11,30,33,34,35,36]. We found high scores of global HRQoL at the time of diagnosis in our study population with locally advanced disease. Nonetheless, there was a significant deterioration in global health status and social functioning with CRT, while symptoms of fatigue and pain increased, as supported by other authors [37,38]. It is important to note that patients maintained high emotional functioning. Some studies also suggest a progressive improvement in emotional functioning after treatment [39].

Patient-reported QoL is an indicator of overall well-being, which can measure self-perceived disabilities related to the disease or treatment. The decline in social functioning was expected since treatment negatively affects the structure and functioning of the head and neck region, which is highly exposed in social interactions. The increased fatigue after treatment is consistent with the results of Jereczek-Fossa et al., who analysed a heterogenous population of HNC patients submitted to radiotherapy and reported a progressive increase in fatigue, with a maximum score on the 6^th^ week of radiotherapy and a slow decrease afterwards [40].

More than 65% of the patients presented at least mild cognitive impairment at pre-treatment assessment, and 50% only knew how to read or write or had primary education. These results are consistent with the literature [41] and highlight the importance of support care and rehabilitation in this cancer population, especially if we take into consideration that cognitive function may influence treatment adherence and outcomes [41,42]. We found no acute deterioration of cognitive function assessed by the EORTC QLQ-C30 questionnaire or by the MoCA test. These results should be interpreted carefully, as it has been reported that radiotherapy has a late effect on neurocognitive function [43].

Muscle strength has an important role in overall functional ability. In our study, HNC patients had a median quadriceps maximal isometric muscle strength of 31.7 kgf in the dominant limb and 30.5 kgf in the nondominant limb before treatment. Both of these values are lower than those reported for chronic obstructive pulmonary disease (34.4 kgf) patients and for healthy individuals (43.8 kgf) [44]. Multiple factors may contribute to this. Low physical activity levels before treatment commencement have been reported in this cancer population [45,46]. Most of our patients reported a weight loss superior to 10% during 6 months prior to diagnosis and were moderately or severely malnourished according to PG-SGA, which may be due to decreased caloric intake or an early manifestation of cachexia [47]. A recent meta-analysis showed that the prevalence of sarcopenia was common in the pre-treatment phase, ranging from 6.6% to 64.6%, which may reflect impaired muscle strength and physical function [48].

Weight loss has prognostic value for overall survival in these patients since it increased treatment-related toxicities and decreased the tolerance and response to treatment [47,49,50]. The correlation between pain and PG-SGA suggests that an early diagnosis and better symptomatic control may prevent the decline of nutritional status. With regard to this matter, clinicians should pay particular attention to radiation-induced oral mucositis, which may occur in about 91% of cases, with these patients being significantly more likely to have severe pain (54% vs. 6%, *p* < 0.001) and a weight loss of ≥5% (60% vs. 17%, *p* < 0.001) [51].

At the end of CRT, all patients were moderately or severely malnourished, and there was a significant loss of body fat and fat-free mass. This was expected, as advanced tumour stage and the use of CRT were suggested to be independent risk factors for weight loss [52]. Moreover, at the end of treatment, these patients often experienced swallowing impairment because of the toxicity profile of CRT. Indeed, in our study, all patients scored higher than 15 points in EAT-10, indicative of a risk of aspiration [53]. This emphasizes the need for close monitoring of swallowing dysfunction throughout the whole of the cancer care continuum. Of note, EAT-10 reflects changes in FOIS during CRT, as corroborated by Ishii et al. [54].

Additionally, it is known that this negative shift in body composition is associated with lower physical performance. Indeed, we observed a significant decline in upper body muscle strength during CRT, but not in lower body muscle strength. Our results for handgrip strength are in line with those reported by Chauhan et al. [55], who observed a significant decrease after CRT when patients used the dominant hand. This may be explained by the increased effect of radiotherapy on the neck and upper body. As reported by other authors [14,56,57], we would expect a significant decrease in 6MWT during CRT, in line with the increased self-reported fatigue symptom scale score and the loss of fat-free mass. Nevertheless, there was consistency in the self-reported (physical functioning subscale) and objective results (6MWT, 30 s STS repetitions, and isometric quadriceps muscle strength) regarding physical functioning. 

ET may be an important coadjuvant therapy to minimize the negative impact of HNC treatment, not only in physical fitness, but also in QoL and cancer-related fatigue, as suggested by the promising results of recent pilot studies [13,14,17,18,56,57]. These results encourage the development of an ET program before treatment, FIT4TREATMENT, a randomized controlled trial design to analyse the potential benefits of ET on HNC patients with locally advanced disease. An ET program immediately after diagnosis can potentially improve physical reserves and overall health, leaving the patient less susceptible to treatment-related complications or functional decline. Moreover, high adherence rates are expected since patients have not yet been exposed to treatment toxicities.

The strengths of this study include the analysis of multiple outcomes reflecting real-world concerns about the management of HNC patients with locally advanced disease, therefore fulfilling a clinical need to better understand this patient population. The prospective design and use of standardized and validated tests allowed a longitudinal and consistent evaluation. One main limitation is the small sample size, especially due to the study’s early suspension due to the COVID-19 pandemic, which compromised the analysis of acute toxicities according to different treatment schemes.

## 5. Conclusions

Newly diagnosed HNC patients are a vulnerable population, often with low socioeconomic status and mild cognitive impairment. In the pre-treatment phase, most patients present low physical function and are moderately malnourished or at risk of malnutrition. Patients subjected to radical CRT experience a significant decline in HRQoL, physical function, swallowing capacity, and nutritional status over the course of the treatment. We expect that the ET program will optimize patients’ physical fitness, improving treatment efficacy with less toxicity.

## Figures and Tables

**Table 1:** Patients’ baseline sociodemographic and disease-related characteristics (n = 18).

Characteristic	Total
Age in years—median (IQR)	53.3 (51.8–65.5)
Gender, male—n (%)	18 (100)
ECOG-PS—n (%) 0 1	7 (38.9) 11 (61.1)
Marital status—n (%) Single/divorced Married	5 (27.8) 13 (72.2)
Education—n (%) ≤Primary education >Primary education	9 (50.0) 9 (50.0)
Employment—n (%) Employed Unemployed Retired	8 (44.4) 5 (27.8) 5 (27.8)
Smoking status—n (%) Current Former smoker ^1^	8 (44.4) 10 (55.6)
Alcohol status—n (%) Never drinker Current drinker Former drinker	1 (5.6) 7 (38.9) 10 (55.6)
Primary tumour location—n (%) Larynx Oral cavity Pharynx	2 (11.1) 7 (38.9) 9 (50.0)
Stage—n (%) III IVA IVB	1 (5.6) 12 (66.7) 5 (27.8)
Treatment—n (%) Induction CT followed by CRT Induction CT followed by surgery ± RT Radical CRT Surgery followed by CRT	2 (11.1) 5 (27.8) 9 (50.0) 2 (11.1)

^1^ Former smoker: previous smoker who quit more than 12 months ago.

**Table 2:** Baseline quality of life, physical fitness, dysphagia, nutritional status, and cognitive function (n = 18).

Characteristic	Total	
	Median	IQR
**HRQoL—EORTC QLQ-C30 (score)**		
Global health status	70.8	50.0–83.3
Functional scales		
Physical functioning	86.7	73.3–100
Emotional functioning	75.0	66.7–85.4
Cognitive functioning	91.7	83.3–100
Social functioning	100	66.7–100
Symptom scales		
Fatigue	11.1	8.3–33.3
Pain	16.7	12.5–50.0
Insomnia	33.3	0–41.7
Appetite loss	0	0–33.3
Financial difficulties	16.7	0–66.7
**HRQoL—EORTC QLQ-HN43 (score)**		
Swallowing	20.8	0–50.0
Dry mouth and sticky saliva	25.0	0–33.3
Body image	5.6	0–22.2
Fear of progression	16.7	16.7–66.7
**Physical function**		
6 min walk test (meters)	434	399–533.8
30 s sit-to-stand test (repetitions)	13.5	12.0–15.5
Isometric handgrip strength (kgf)		
Dominant hand	38.0	34.7–44.0
Nondominant hand	37.1	33.7–41.3
Isometric quadriceps strength (kgf)		
Dominant limb	31.7	20.7–36.5
Nondominant limb	30.5	20.9–35.6
Nutritional status and body composition *		
Body mass index (kg/m^2^)	21.9	18.0–25.1
Global Assessment PG-SGA (score)	12.0	7.0–16.0
**Cognitive function**		
MoCA (points)	23	20.8–26.3
Dysphagia *		
EAT-10 total score (points)	7.5	1.8–22.3
FOIS score (points)	5.0	5.0–6.0

* One patient did not complete baseline nutritional and dysphagia evaluations.

**Table 3:** Acute impact of CRT on quality of life, physical fitness, nutritional status, dysphagia and cognitive function (n = 7).

	N	Baseline T0	Post-Treatment T1	*p*-Value	Effect Size
**HRQoL—EORTC QLQ-C30 (score) – median (IQR)**					
Global health status	7	75 (66.7–83.3)	50 (33.3–66.7)	0.014 ^1^	0.657
Functional scales	7				
Physical functioning		86.7 (60–100)	80 (73.3–86.7)	0.684	0.109
Emotional functioning		75 (66.7–83.3)	77.8 (58.3–83.3)	1.000	0.000
Cognitive functioning		100 (83.3–100)	100 (83.3–100)	0.414	0.218
Social functioning		100 (100–100)	66.7 (66.7–100)	0.046 ^1^	0.567
Symptom scales	7				
Fatigue		11.1 (0–33.3)	44.4 (33.3–77.8)	0.026 ^1^	0.595
Pain		16.7 (16.7–33.3)	33.3 (33.3–50)	0.038 ^1^	0.553
Insomnia		0 (0–33.3)	66.7 (0–66.7)	0.063	0.496
Appetite loss		0 (0–0)	0 (0–33.3)	0.157	0.378
Financial difficulties		33.3 (0–66.7)	66.7 (0–100)	0.414	0.218
**HRQoL—EORTC QLQ-HN43 (score) – median (IQR)**					
Swallowing	6	29.2 (0–52.1)	66.7 (25–72.9)	0.043 ^1^	0.584
Dry mouth and sticky saliva	6	0 (0–33.3)	66.7 (45.8–87.5)	0.041 ^1^	0.589
Body image	7	0 (0–11.1)	11.1 (0–55.6)	0.109	0.429
Fear of progression	7	16.7 (16.7–66.7)	33.3 (16.6–33.3)	0.785	0.073
**Physical function – median (IQR)**					
6MWT (meters)	7	486 (412–533)	422 (362–510)	0.236	0.317
30 second sit-to-stand test (reps)	6	13 (12–17)	14 (12–17)	0.833	0.061
Isometric handgrip strength (kgf)					
Dominant hand	7	39.7 (35.0–50.0)	35.0 (31.7–39.3)	0.018 ^1^	0.632
Nondominant hand	7	37.2 (33.6–42.5)	33.9 (34.3–41.2)	0.043 ^1^	0.542
Isometric quadriceps strength (kgf)					
Dominant limb	7	33.8 (26.3–36.5)	28.7 (25.7–31.4)	0.176	0.361
Nondominant limb	7	30.8 (22.4–37.0)	26.2 (19.3–33.4)	0.237	0.316
**Nutritional status and body composition**					
BMI (kg/m^2^) – median (IQR)	7	24.2 (18.1–25.6)	20.6 (18.6–22.7)	0.028 ^1^	0.587
Body fat (%) – median (IQR)	6	21.1 (11.3–26.4)	15.3 (11.8–20.3)	0.046 ^1^	0.575
Fat-free mass (kg) – n (%)	6	51.8 (46.0–55.5)	49.0 (44.1–52)	0.046 ^1^	0.575
Global Assessment PG-SGA	6				
A. Well nourished		3 (50%)	0 (0%)	0.549	
B. Moderately malnourished or suspected malnutrition		2 (33.3%)	1 (16.7%)		
C. Severely malnourished		1 (16.7%)	5 (83.3%)		
PG-SGA total score (points)	6	7 (3–13)	18 (15–23)	0.028 ^1^	0.635
**Dysphagia**					
EAT-10 total score (points) – median (IQR)	7	7 (0–11)	31 (21–40)	0.027 ^1^	0.590
FOIS score – n (%)	7				
Nothing by mouth		1 (14.3%)	2 (28.6%)	0.203	
Tube dependent with minimal attempts of food or liquid		0 (0%)	1 (14.3%)		
Total oral diet with multiple consistencies, but requiring special preparation or compensations		2 (28.6%)	1 (14.3%)		
Total oral diet with multiple consistencies without special preparation, but with specific food limitations		2 (28.6%)	2 (28.6%)		
Total oral diet with no restrictions		2 (28.6%)	1 (14.3%)		
FOIS total score (points)	6	6 (5–7)	4 (1–5)	0.041 ^1^	0.545
**Cognitive function**					
MoCA (points) – median (IQR)	7	26 (20–27)	23 (20–26)	0.167	0.369

^1^ Significant results (*p*-value < 0.05).

## Data Availability

The data presented in this study are available on request from the corresponding author. The data are not publicly available due to privacy and ethical reasons.
